# Deep venous thrombosis Doppler screening in critically ill patients: is it justified?

**DOI:** 10.1186/cc9435

**Published:** 2011-03-11

**Authors:** I Vlachou, G Petrocheilou, E Evodia, M Pappa, L Livieratos, P Myrianthefs, L Gregorakos, G Baltopoulos

**Affiliations:** 1 St Paul Hospital, Athens, Greece; 2 Agioi Anargyroi Hospital, Athens, Greece

## Introduction

The purpose of this study was to determine the incidence of asymptomatic deep venous thrombosis (DVT) in long-stay critically ill patients.

## Methods

Over an 8-month period, 53 patients were admitted and anticipated to stay in the ICU for >48 hours. DVT prophylaxis was provided using low molecular weight heparin (LMWH) or a sequential leg compression device as medically indicated. Patients had a baseline Duplex Ultrasound Screening (DUS) examination on admission and screening on a weekly basis regardless of clinical or laboratory evidence for DVT. Demographics and ultrasound data were also collected.

## Results

We studied 53 patients (42 males, mean age (SEM) 57.6 (2.8) years, illness severity scores APACHE II 21.3 (0.9); SAPS II 53.3 (2.3); SOFA 10.2 (0.2); and ICU stay 35.9 (4.8) days). Eleven (20.8%) of them developed DVT on day 7.4 (1.8), on DUS. Six patients had lower limb DVT, five upper limb DVT. Another one had DVT on admission. In group A (Table 1), six patients (37.5%) developed DVT on day 7.0 (2.4) without receiving LMWH due to underlying disease (hemorrhagic stroke, brain injury), but only pneumatic compression. In group B (Table 1), five patients (13.5%) developed DVT on day 7.7 (2.9) despite timely and appropriate LMWH administration since ICU admission. None of the patients in both groups developed pulmonary embolism. The difference regarding the incidence between the two groups was statistically significant (*P *= 0.042, RR: 2.847 (CI: 1.050 to 7.721), OR: 4.167 (CI: 0.989 to 17.55)).

**Table 1:** 

	Group A	Group B	*P *value
*n *(patients)	6/16	5/37	0.042
APACHE II	25.8 (3.3)	21.8 (2.1)	0.49
SAPS II	55.5 (6.3)	66.4 (9.8)	0.55
SOFA	10.5 (1.2)	11 (2.19)	1.0
Day DVT	7.0 (2.4)	7.7 (2.4)	0.36
LOS ICU	60.2 (37)	71.2 (39)	0.53

## Conclusions

According to our results the application of DUS screening in ICU patients seems to be justified for early, accurate diagnosis of silent DVT and appropriate therapy.

